# Influence of Butter Layer Thickness on Microstructure and Mechanical Properties of Underwater Wet 16Mn/304L Dissimilar Welded Joint

**DOI:** 10.3390/ma16206646

**Published:** 2023-10-11

**Authors:** Ke Han, Yunhu Cao, Hongliang Li, Chengyu Hu, Zeyu Wang, Duo Liu, Jianfeng Wang, Qiang Zhu

**Affiliations:** 1 School of Materials Science & Engineering, Jiangsu University, Zhenjiang 212013, China; hanke@ujs.edu.cn (K.H.);; 2 State Key Laboratory of Advanced Welding and Joining, Harbin Institute of Technology, Harbin 150001, China; 3 College of Materials Science and Technology, Nanjing University of Aeronautics and Astronautics, Nanjing 211106, China; wangjianfeng@nuaa.edu.cn

**Keywords:** underwater wet welding, dissimilar welded joint, microstructure, mechanical properties, butter layer

## Abstract

Butter layers of different thicknesses were successfully deposited on ferritic steel by using the tungsten arc welding (TIG) process. The effects of butter layer thickness on the microstructural characteristics, elemental distribution, and mechanical properties of underwater wet 16Mn/304L dissimilar welded joints were investigated. The results showed that the butter layer significantly changed the microstructure and elemental distribution of 16Mn/304L joints. As the thickness of butter increased, the heat-affected zone (HAZ) at the ferritic steel side changed from the original 16Mn steel to the ERNiCrMo-3 butter layer. The martensite content in HAZ also exhibited a downward trend. When the thickness of the butter layer exceeded 6 mm, the microstructure of HAZ at the ferritic steel side was composed of ferrite and pearlite, instead of quenched martensite. The microhardness of underwater dissimilar steel welded joints significantly reduced due to the absence of martensite. The addition of the butter layer increased the ultimate tensile strength from 515 MPa to 565 MPa. The results of this work could provide a robust basis for future applications of dissimilar steel structures.

## 1. Introduction

Austenitic stainless steel 304L is widely used in corrosion-resistant pipelines, marine propellers, and pressure vessels, due to its superior tensile strength and corrosion resistance. The high content of alloying elements in 304L steel drives up production costs. The low alloy steel, 16Mn, which is characterized by a low carbon content and the presence of few alloying elements, possesses desirable qualities such as high strength, exceptional toughness, and cost effectiveness. It is valuable to replace some 304L steel structural components with 16Mn steel in less corrosive situations. Therefore, dissimilar steel joints between 16Mn and 304L are extensively employed in the various applications such as offshore wind power plants, oil and gas pipelines, and offshore platforms. These dissimilar steel structural parts are vulnerable to detrimental effects of working loads, wind/waves, and ocean currents. These factors might potentially result in the development of cracks, ultimately leading to structural damage. The development of underwater welding technology becomes imperative for the installation and replacement of dissimilar steel components.

Underwater welding technology is mainly divided into three types: dry welding, local dry welding and wet welding. As suggested by Tomków et al. [1], the dry welding process with relatively huge costs requires building special chambers for dry welding. The underwater local dry welding procedure was highly appealing, due to its ability to achieve high-quality weld and keep costs controlled. Underwater laser welding [2], metal inert gas welding (MIG) [3], and tungsten inert gas welding (TIG) [4] were widely studied alongside local dry welding technologies. In terms of weld quality and cost, the underwater local TIG welding process is the most advantageous option. However, high-quality underwater TIG welds are mainly applied when joining vital structures, because of the low welding efficiency. In addition, underwater friction welding was proposed to avoid problems associated with arc welding processes [5], but joint geometry restricted its application. To date, underwater wet welding is still widely used in offshore structures, owing to its convenience and cost-effectiveness.

Compared with the welding of similar materials, joints between low-alloy steel and austenitic stainless steel displayed notable disparities in their thermal-physical properties, including thermal conductivity and coefficient of thermal expansion [6]. The alloying elements in the welds were severely diluted by the base metal (BM), due to substantial compositional differences between dissimilar steels [7]. The above phenomenon led to the heterogeneous distribution of the microstructural characteristics, element distribution and mechanical properties adjacent to the fusion boundary [8]. Moreover, when welding and during the subsequent service of low-alloy steel/austenitic stainless steel welded joints, carbon migration from the low-alloy steel to the austenitic weld metal occurred, causing the formation of a carbon-depleted region on the low-alloy steel side [9]. Meanwhile, martensite was formed on the austenitic weld metal side between the type-II boundaries and fusion boundary, which hardened the joint but reduced its ductility and toughness [10]. The dissimilar steel welded joints (DSWJ) experienced a significant stress concentration due to the substantial difference in their coefficients of thermal expansion. As a result, DSWJ exhibited an obvious heterogeneity in terms of chemical composition, microstructure, performance, and stress distribution. Extensive research has been conducted by scholars to attain superior DSWJs. This research encompassed diverse areas, including optimization of the welding process parameters [11,12], control of the structure-property [13,14], mitigation of residual stress [15], and the utilization of butter layers [16].

Apart from the above issues related to dissimilar steel welding, the adverse effects of a water environment should also be considered during the underwater wet welding of dissimilar steel. The presence of water considerably changed the solidification process of the molten pool, welding process stability, microstructural evolution and properties, elemental diffusion, and the stress distribution of dissimilar steel joints [17]. Some relevant studies have been conducted on the underwater wet dissimilar steel welded joints. Tomków et al. pointed out that temper bead welding techniques could be used to mitigate the quenching tendency in the HAZ of low-alloy high-strength steel [18]. The development of austenitic-type consumables with high quality was critical to obtain superior underwater wet DSWJ, as suggested by Guo et al. [19].

Previous work in our research group reported that 16Mn/304L dissimilar steel could be successfully underwater wet welded with specially developed nickel-based self-shielded flux-cored wires [20]. Nevertheless, the dissimilar steel welded joint exhibited a rather low level of comprehensive performance. The performance of the joint was hampered by the lower toughness of the coarse-grained HAZ on the low-alloy steel side. Further research indicated that the introduction of a butter layer served multiple purposes in the onshore welding of dissimilar steels, including low-alloy steel and austenitic stainless steel. The advantages [21,22,23,24] included mitigation of martensitic transformation in welded joints, reduction of residual stresses near the fusion boundary on the low-alloy steel side, decrease of the fatigue crack propagation rate, enhancement of the joint’s strain-hardening capability, and effective prevention of carbon diffusion into the weld metal. Consequently, these benefits led to an improvement in the weldability of dissimilar steel. The prepared butter layer could effectively improve the tensile strength and impact toughness of dissimilar steel joints if this technique was applied in an underwater environment [25]. However, the existing research has yet to elucidate the influence of interlayer thickness on microstructure and mechanical properties of underwater wet DSWJ.

Therefore, the present investigation proceeded with the practice of pre-placing butter layers with different thicknesses on the side of low-alloy steel. The effects of butter layer thickness on microstructural characteristics and mechanical properties of underwater DSWJ were analyzed. The goal was to determine the most effective thickness of buttered layer that could improve the performance of dissimilar welded joints. The results of this work can provide a robust basis for future applications of dissimilar steel structures.

## 2. Experimental Procedures

Low-alloy steel of 16Mn and austenitic stainless steel of 304L were selected as the BMs, with the former supplied in a cold-rolled state and the latter subjected to solid solution treatment. The dimensions of the specimens were 200 mm × 60 mm × 6 mm. The dissimilar welds were manufactured using specially formulated nickel-based tubular wire. The slag system consisted of CaF_2_-Al_2_O_3_-MgO with a slag basicity of 2.66. The underwater wet self-shielded flux-cored wire arc welding process was employed to achieve the welding of dissimilar steels at a water depth of 0.5 m. The welding parameters utilized in this study were as follows: arc voltage of 30 V, wire feeding speed of 4.5 m/min, welding speed of 120 mm/min, and contact tip-to-work distance of 20 mm. An AoTai Pulse MIG-350 power source with DCEP polarity was used in the experiments. In order to achieve complete penetration and the desired backside formation, a backing plate made of copper with a thickness of 3 mm was employed. This backing plate was equipped with an arc-shaped groove that was 1.5 mm deep and aligned with the center of the weld bead.

**Figure 1:**
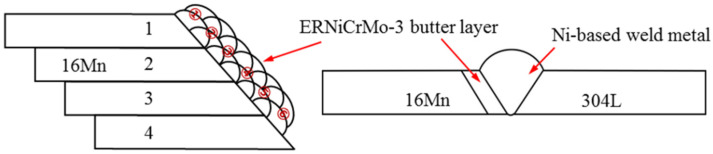
Schematic diagram of deposited butter layer at 16Mn side and dissimilar steel welded joint with butter layer.

In this experiment, a Fronius MagicWave 3000 power source was employed to prepare butter layers with various thicknesses on the machined groove of the 16Mn steel in the air. Commercially obtained ERNiCrMo-3-type wire with a diameter of 1.2 mm was selected as the filler material. The Tungsten Inert Gas (TIG) welding process adopted a multi-layer and multi-pass welding technique, wherein the thickness of the butter layer was controlled by adjusting the number of welding passes. The main depositing parameters were listed as follows: welding current of 140 A, wire feeding speed of 0.5 m/min, welding speed of 150 mm/min, and gas flow of 15 L/min. The main steps in depositing the butter layer were as follows: (1) Four pieces of 16Mn low-alloy steel were fixed together by TIG spot welding process; (2) The first ERNiCrMo-3 layer with a thickness of 2 mm consisted of six weld passes positioned adjacent to one another at an overlap rate of 50%, as depicted in Figure 1; (3) Air cooling before sequentially welding the latter layer. The temperature of the interlayer was maintained within the range of 40–60 °C; (4) After cladding, the joints were separated by wire cutting and the butter layer was machined to achieve the thickness of 2 mm from the interface of 16Mn steel to ERNiCrMo-3 butter layer; (5) Repeated steps (1)–(4) to manufacture the second, third and the fourth layer. Underwater wet welding was carried out with a butter layer of different thicknesses (2 mm, 4 mm, 6 mm, and 8 mm) on the groove surface of 16Mn.

As-received welds were sectioned for microstructural analysis and mechanical properties tests. Metallographic samples were produced in accordance with the GB/T13298-2015 standard [26]. The microstructure of 16Mn BM and HAZ, the 16Mn steel/Ni-based weld metal interface, and the 304L/Ni-based weld metal interface was revealed using 4% nitric acid solution, 10% oxalic acid, and HCl+HNO_3_, respectively. The microstructure was observed using an optical microscope and scanning electron microscope equipped with an Energy Dispersive Spectrometer (EDS). The grain boundary type and grain orientation across the interface of 16Mn/butter layer (weld metal) were also analyzed, using electron backscattering diffraction (EBSD). At room temperature, transverse tensile test was conducted to determine the ultimate tensile strength of underwater DSWJ. Three samples at least were evaluated for each condition. The Vickers microhardness was determined by applying a load of 2.942 N for 10 s across the welded joints. Lastly, the micro-fracture surfaces of welded joints were examined using the SEM.

## 3. Results and Discussion

### 3.1. Microstructure of the Deposited Butter Layer

**Figure 2:**
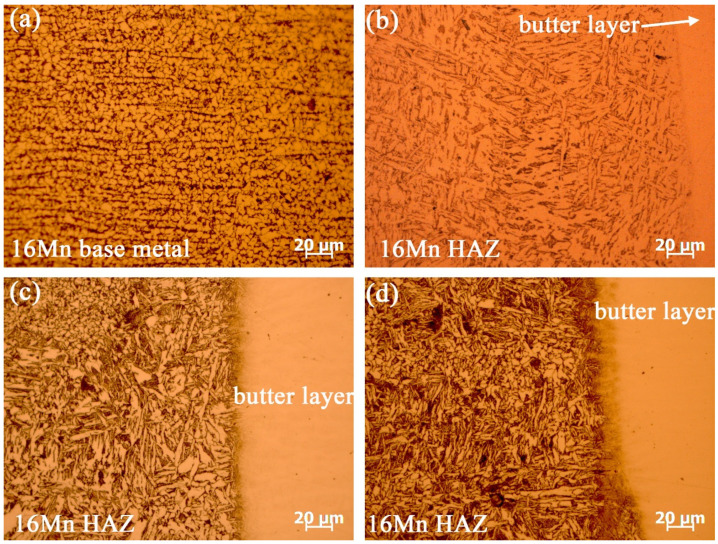
The effect of butter layer thicknesses on the microstructure of 16Mn HAZ (**a**) 16Mn steel base metal without butter layer, (**b**) 2 mm, (**c**) 6 mm, (**d**) 8 mm.

Prior to underwater wet welding, a thorough examination of microstructural evolution and elemental distribution of the deposited butter layer with ERNiCrMo-3 filler on low-alloy steel 16Mn was performed. Figure 2 displays the microstructural evolution at the fusion boundary after introducing butter layers with different thicknesses. The microstructure of 16Mn steel in Figure 2a exhibits a typically rolled microstructure with ferrite and pearlite. Figure 2b illustrates the presence of coarse ferrite with a percentage of pearlite in HAZ after the application of a 2 mm-thick butter layer. When the thickness of the butter layer increased to 6 mm, the ferrite plate in HAZ exhibited evident reduction in length and width, as shown in Figure 2c. As the thickness of butter layer reached 8 mm, the ferrite in HAZ was further refined, due to the subsequent weld thermal cycles. Simultaneously, a distinct microstructural transition region was observed at the fusion boundary and the width of the transition zone expanded with the increase of the deposited layer thickness.

**Figure 3:**
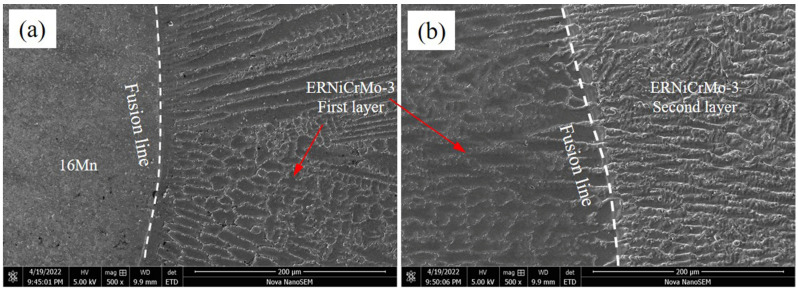
Microstructure of TIG surfacing layer on 16Mn steel side (**a**) across the fusion boundary 16Mn side, (**b**) the area between nickel base butter layer.

Figure 3 shows the SEM image of the TIG surfacing layer on the 16Mn steel side in the air. Evidently, a transition zone with a width of approximately 20 μm existed at the fusion boundary owing to the compositional changes. Type-II grain boundaries parallel to the fusion boundary were observed between the butter layer and 16Mn steel. The formation of Type II grain boundaries were a result of grain boundary migration within the austenite temperature range during solidification process [27]. The butter layer was fully austenitic with well-developed columnar sub-grains. The grains tended to grow along the direction of the maximum thermal gradient, which was perpendicular to the fusion line. As shown in Figure 3b, an interface was formed between the previous butter layer and the succeeding layer. The excellent fusion of butter layers indicated that the butter layers prepared by the TIG process were acceptable. The microstructure near the fusion line between adjacent layers varied little. The columnar grains across the interface between butter layers predominantly grew epitaxially from the previous butter layer grains.

**Figure 4:**
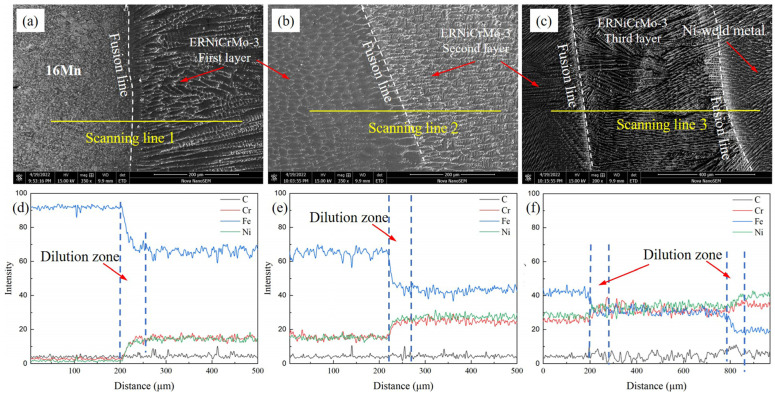
Elemental distribution in the butter layer with a thickness of 8 mm (**a**,**d**) 16Mn/ERNiCrMo-3 layer, (**b**,**e**) the first butter layer/the second layer, (**c**,**f**) the second layer/the third layer/Ni-based weld metal.

Elemental variations in the different regions, after cladding the ERNiCrMo-3 butter layer, were analyzed using EDS analysis, and the results are illustrated in Figure 4. Figure 4a demonstrates that Fe content in 16Mn BM was considerably higher than that in the nickel-based butter layer, whereas Ni and Cr content were relatively lower. A diluted zone with the width of about 50 µm was formed at the interface. During the multi-layer and multi-pass TIG cladding process, the dilution effect of subsequent layers on the previous layer resulted in a gradual decrease in Fe content and a gradual increase in Cr and Ni content as the number of layers increased. Figure 4b shows the fusion boundary between the first two butter layers, where an insufficiently mixed transition zone with a width of approximately 30 μm existed. When the number of butter layers exceeded three, the content of Cr and Ni elements in the fusion zone showed little variation and approached that of the ERNiCrMo-3 filler metal, while Fe content decreased gradually until it reached equilibrium with the composition of the Ni-based weld metal, as depicted in Figure 4c.

### 3.2. Microstructural Characteristics of Underwater DSWJ

**Figure 5:**
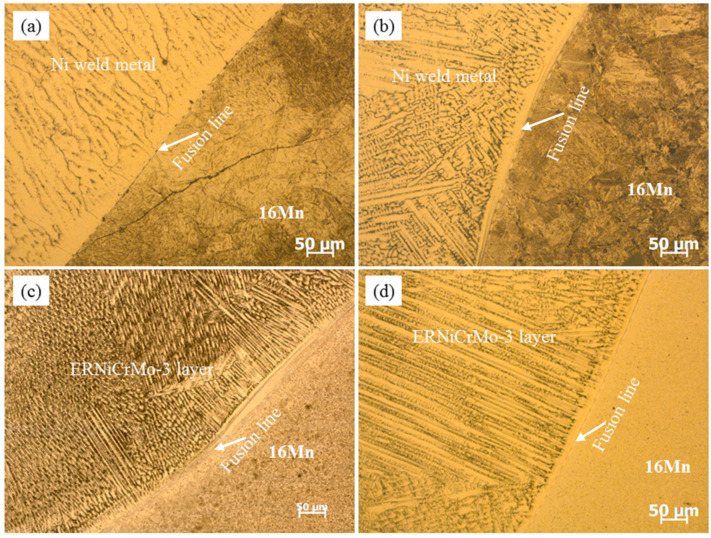
Microstructure at 16Mn side for the underwater DSWJ with different layer thickness (**a**–**d**) where the butter layer thickness was 2 mm, 4 mm, 6 mm and 8 mm, respectively.

Figure 5 illustrates the optical microstructure across the fusion boundary on the 16Mn side with different butter layer thicknesses. When the thickness of the butter layer was 2 mm, it was fully melted during the underwater welding process and no isolation effect was observed. As displayed in Figure 5a, a significant quantity of coarsened martensite was present within the HAZ on the 16Mn side. The susceptibility of this region to hydrogen-induced cracking (HIC) was high, which could be verified by the formation of a crack in the coarsened-grain HAZ. When the butter layer thickness was 4 mm, the HAZ width at 16Mn side noticeably reduced, although lath martensite still existed in Figure 5b. When the butter layer thickness exceeded 6 mm, HAZ at the 16Mn steel side transferred from the original 16Mn BM to the ERNiCrMo-3 butter layer in Figure 5c,d, resulting in the disappearance of martensite in HAZ on 16Mn steel side.

**Figure 6:**
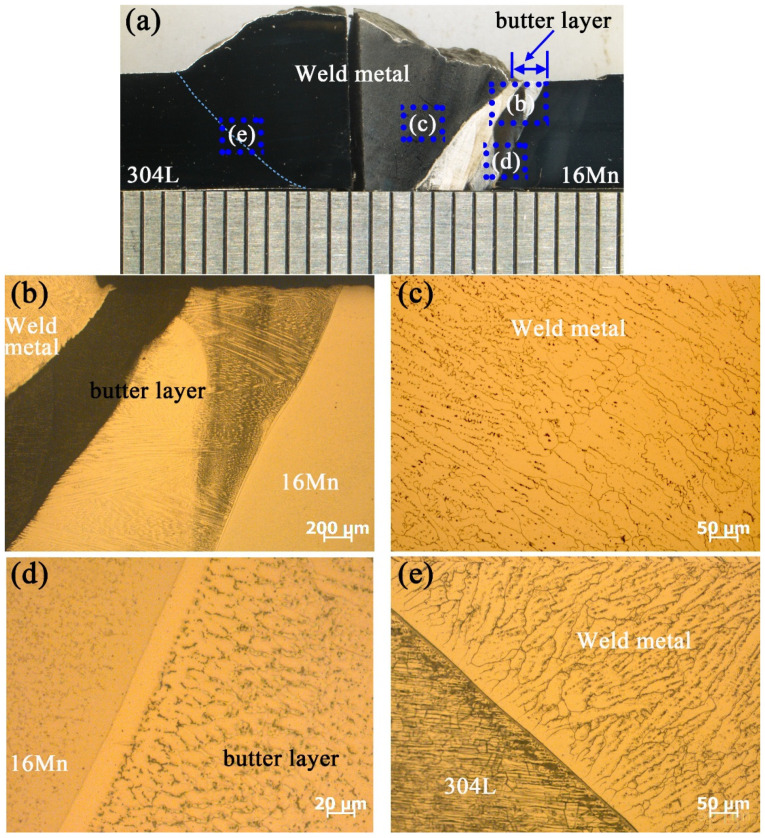
The typical microstructure of underwater DSWJ with 8 mm-butter layer (**a**) Macroscopic image of underwater DSWJ, (**b**) Microstructure of ERNiCrMo-3 butter layer, (**c**) nickel-based weld metal, (**d**) 16Mn HAZ/butter layer interface, (**e**) 304L HAZ/Ni-based weld metal.

Figure 6 presents the microstructure of underwater wet DSWJ with a butter layer of 8 mm. The typical cross-section of the underwater DSWJ is displayed in Figure 6a, which reveals the existence of approximately 3~4 mm thick interlayers adjacent to 16Mn steel. Pores and cracks were not detected in the welded joints. Figure 6b shows a low-magnification image of ERNiCrMo-3 butter layer. A part of the butter layer was melted into the Ni-based welds, which resulted in a reduction of the butter layer thickness from 8 mm to 4 mm. A corrosion-resistant layer existed between the nickel-based weld and the unmelted butter layer. As displayed in Figure 6c, the nickel-based weld metal exhibited a fully austenitic structure with columnar features. At the interface between the 16Mn and ERNiCrMo-3 interlayers, a transition zone with a width of 30 μm could be observed in Figure 6d, which was consistent with the elemental changes after depositing the butter layer on the 16Mn side. Importantly, HAZ on the 16Mn steel side was entirely composed of coarse ferrite grains and pearlite, instead of hardened lath martensite. The residual stresses and HIC tendency in the coarse-grained HAZ were mitigated. The microstructure at the side of 304L steel is depicted in Figure 6e, where HAZ with a width of 150 µm was discerned. The weld metal displayed evident epitaxial growth at the interface and the HAZ contained δ-Fe ferrite generated within or along the boundaries of coarse austenite grains.

### 3.3. Elements Distribution of Underwater DSWJ

**Figure 7:**
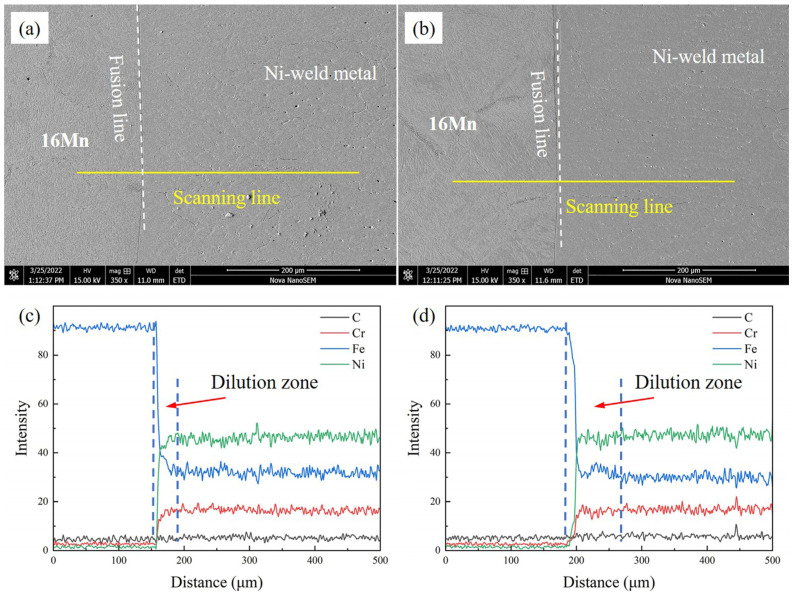
Elemental distribution across the FB in underwater DSWJ with different butter layer thicknesses (**a**,**c**) 2 mm, (**b**,**d**) 4 mm.

**Figure 8:**
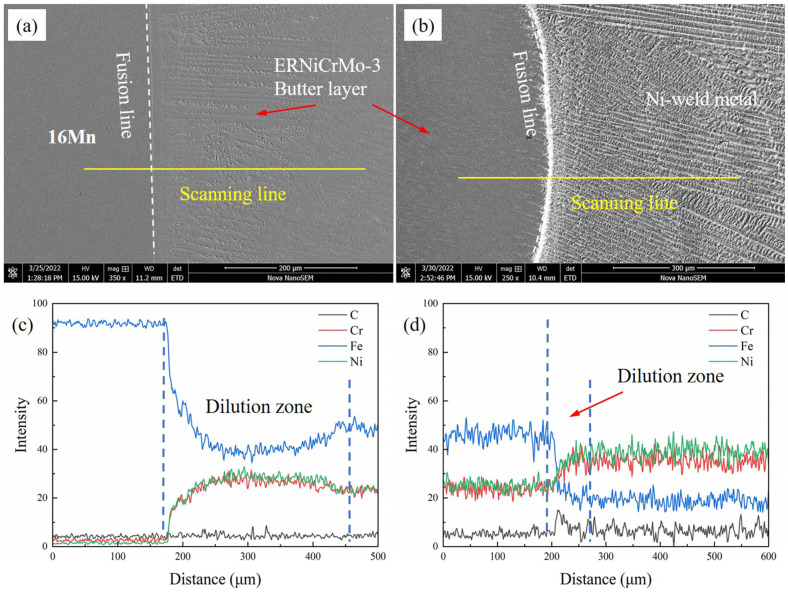
Elemental distribution across the FB at a layer thickness of 8 mm (**a**,**c**) the interface between 16Mn and butter layer, (**b**,**d**) the interface between ERNiCrMo-3 layer and nickel-based weld.

The chemical composition distribution of underwater DSWJ showed significant changes due to the intricate welding heat cycles and the compositional differences between the filler metal and BMs. The line scanning results across the 16Mn steel/butter layer interface with different layer thicknesses are depicted in Figure 7 and Figure 8a. These curves, obtained at different butter layer thicknesses, exhibited similar patterns. Obvious fluctuation of Cr, Fe, and Ni elements from 16Mn steel to nickel-based weld metal across the interface were found. The Fe content decreased significantly while Ni and Cr content gradually increased. The variation in composition was caused by the dilution effect of 16Mn steel. For the 2 mm-thick butter layer, the entire butter layer melted into the Ni-based weld metal due to the high heat input during the underwater wet welding process. The Ni-based weld metal obtained with a butter layer was less diluted by 16Mn steel, and a transition zone with a width of approximately 50μm was formed near the fusion boundary. Compared with the underwater wet DSWJ without a butter layer, the weld metal obtained with a butter layer exhibited lower Fe content and higher Ni and Cr content in the transition zone, as depicted in Figure 7a,c. In contrast to the deposited layer in Figure 4a,d, significant changes in chemical composition were observed in the nickel-based weld metal with reduced Fe content and increased Cr and Ni content. After adopting a 4 mm-thick butter layer, that butter layer also completely melted into the weld metal and no residual butter layer was macroscopically seen. Meanwhile, a transition zone of approximately 50μm was also found, as shown in Figure 7b,d.

When the thickness of butter layer was over 6 mm, the butter layer in the dissimilar steel joint could be observed. From Figure 8a,b, an elemental transition zone near the fusion boundary existed from 16Mn steel to the ERNiCrMo-3 layer. Fe content decreased while Cr and Ni content gradually increased in the transition zone. The higher nickel content in the weld metal enhanced the stability of austenite phases, thereby reducing the probability of martensite transformation. A slight concentration gradient of Fe, Cr, and Ni elements can be observed from the ERNiCrMo-3 interlayer towards the nickel-based welds. It was mainly due to the similar chemical composition between the interlayer and the Ni-based weld metal.

**Figure 9:**
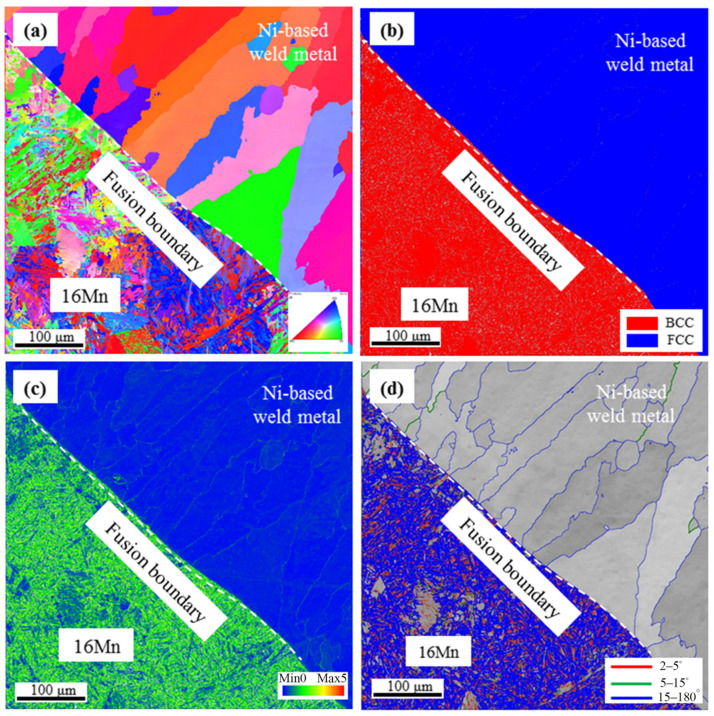
EBSD analysis across the 16Mn steel/Ni-based weld metal with a layer thickness of 2 mm. (**a**) IPF image, (**b**) phase distribution image, (**c**) KAM results, (**d**) grain boundary type diagram.

Figure 9 shows the EBSD results across the interface in underwater DWSJ with a butter layer of 2 mm. From the Inverse pole figure (IPF) image in Figure 9a, the columnar grains in nickel-based weld metal were distributed along the direction of the heat flow, indicating that the preferred orientation occurred in the weld zone. Discontinuous type-II boundaries were found in the nickel-based weld metal adjacent to the fusion boundary. In comparison with underwater DSWJ without a butter layer, the grain size in the weld metal obtained with a 2 mm-thick butter layer was decreased. According to the phase distribution in Figure 9b, a clear boundary between the nickel-based welds and 16Mn steel was observed, due to the face-centered cubic austenite in the weld metal and BCC structure in the HAZ of 16Mn steel. The Kernel Average Misorientation (KAM) analysis results in Figure 9c indicated that a higher level of residual strain existed in the HAZ of 16Mn steel. The result was similar to that of the underwater DSWJ without a butter layer. This was mainly attributed to the complete melting of the 2 mm-thick butter layer during the underwater wet welding process. The lath martensite in the HAZ of 16Mn side was still generated. Type II and type I boundaries in Figure 9d were mostly high-angle grain boundaries (HAGBs) with few low angle grain boundaries (LAGBs). According to the previous investigation, HAGBs with high grain boundary energy would increase corrosion resistance [17]. No significant difference in grain growth characteristics was observed for the joints with a 2 mm-thick butter layer compared with underwater wet DSWJ without a butter layer. Based on the analysis, the butter layer should be increased to at least 6 mm in thickness [25].

### 3.4. Mechanical Properties of Underwater DSWJ

**Figure 10:**
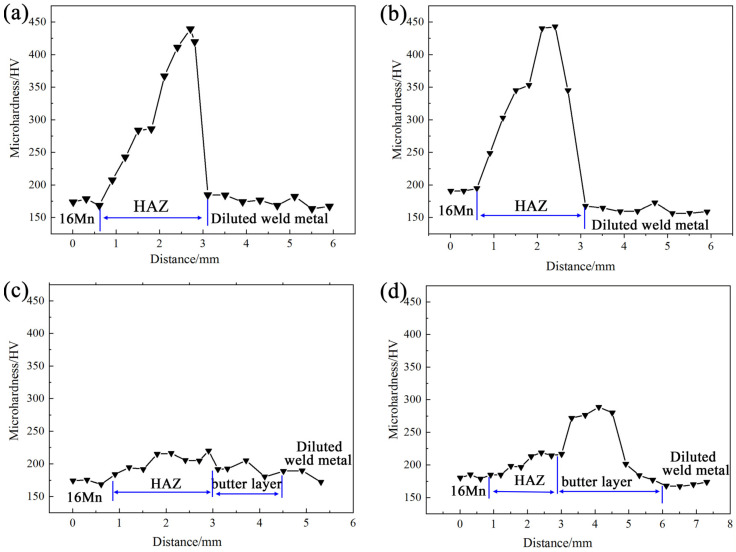
Microhardness across the interface of 16Mn steel/butter layer at different layer thicknesses (**a**) 2 mm, (**b**) 4 mm, (**c**) 6 mm, (**d**) 8 mm.

Microhardness distribution of HAZ on the 16Mn side for underwater wet DSWJ with different butter layer thicknesses is presented in Figure 10. The deposition of butter layers obviously changed the micro-hardness profiles across the interface of 16Mn steel/ butter layer. As the thickness of the butter layer increased, the width of HAZ on the 16Mn side tended to decrease, and the highest micro-hardness shifted from the HAZ of 16Mn side to the ERNiCrMo-3 butter layer. The micro-hardness value of the diluted nickel-based weld metal was approximately 165 HV. For the butter layer with a thickness of 2 mm, a martensite region existed in the coarsened-grain HAZ with a maximum hardness of around 450 HV. When the thickness of the butter layer was 4 mm, because the butter layer was diluted by the weld metal and the isolation effect of the layer disappeared, the quenching was still found. Further increasing the thickness of butter layer to 6 mm or more, the HAZ at the 16Mn side completely transferred to the nickel-based butter layer. The martensite zone in the HAZ of 16Mn then disappeared and the micro-hardness of the whole joint reduced. Notably, the as-deposited ERNiCrMo-3 butter layer with a width of 2 mm exhibited a higher hardness than other regions for joints obtained at a layer thickness of 8 mm.

**Figure 11:**
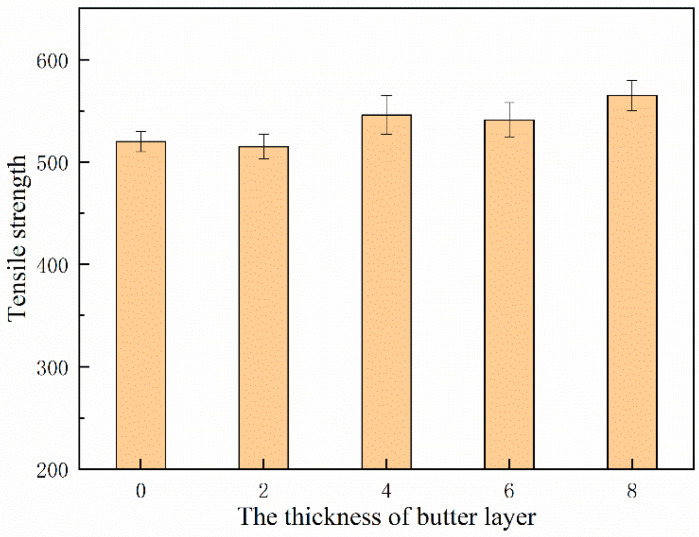
Tensile strength of underwater wet DSWJ with different layer thicknesses.

**Figure 12:**
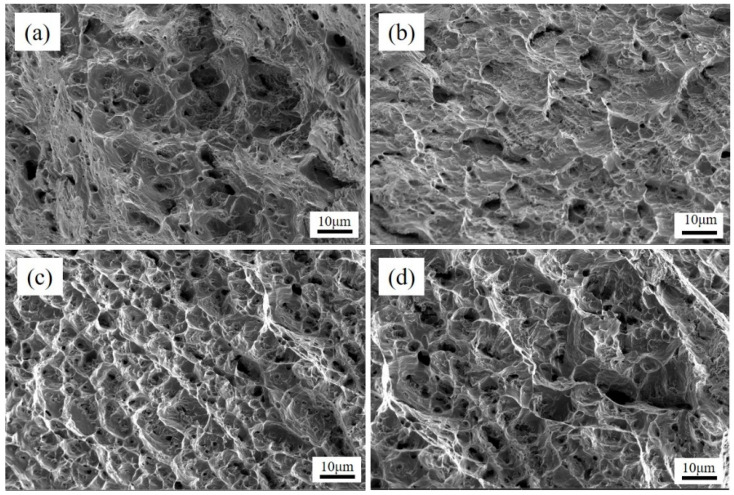
The tensile fracture of specimens with different layer thickness (**a**) 2 mm, (**b**) 4 mm, (**c**) 6 mm, (**d**) 8 mm.

The ultimate tensile strength of underwater wet DSWJ with different layer thicknesses were determined and the results were shown in Figure 11. All specimens fractured either in the nickel-based weld metal or 16Mn BM. A significant plastic deformation occurred prior to fracture, which indicated the high strength of DSWJ with the ERNiCrMo-3 interlayer. The ultimate tensile strength of the specimens was improved due to the introduction of a butter layer. When the specimens fractured in the 16Mn BM, the tensile strength of the DSWJ exceeded 560 MPa. It implied that the nickel-based weld metal and interlayer were higher than that of 16Mn steel. Additionally, the previous studies indicated that the austenitic structure of nickel-based weld metal could be enhanced through the incorporation of alloying elements such as chromium (Cr), iron (Fe), and molybdenum (Mo) [28]. Recalling that the concentration of alloying elements in the weld metal increased as the layer thickness increased. It meant that the solid-solution forming elements increased the strength of the solid solution by primarily increasing the resistance to the movement of dislocations. Subsequently, the weld metal was strengthened by introducing the ERNiCrMo-3 butter layer. Figure 12 displays the SEM images of the fracture surfaces of underwater DSWJ with different layer thicknesses. The fracture morphologies of DSWJ under different butter layer thickness were composed of a large number of dimples. The results indicated that the specimens underwent plastic deformation under tensile loading due to the existence of the higher nickel content in the weld metal. When the thickness of butter layer increased from 2 mm to 8 mm, the size of dimples became smaller and the depth became shallower.

Table 1 lists the Charpy impact toughness of underwater DSWJ at different butter layer thicknesses. Notably, the butter layer had a significant effect on the impact toughness of underwater dissimilar welded joints. The impact toughness of ERNiCrMo-3 butter layer was approximately 100 J/cm^2^. The impact toughness of weld metal was improved due to the introduction of butter layer. This was mainly attributed to the increased alloying elements in buttered weld metal. More importantly, the butter layer obviously reduced the impact toughness of HAZ at the 16Mn side when the butter layer was applied. The impact toughness at HAZ of the 16Mn side was two times less than that of the case when there was no butter layer, which indicated that the HAZ at 16Mn side had a low susceptibility to cold crack.

### 3.5. Advantages of Buttering Layer in Underwater Wet Welding Process

Based on the aforementioned findings, the butter layer on the low-alloy steel side enhanced the comprehensive properties of DSWJ. However, due to the huge challenges in preparing a butter layer on the ferritic steel side in the conditions of underwater on-site repairs, the practical possibility of the TIG depositing technique was low. Meanwhile, the butter layer deposited on the ferritic steel is more difficult to machine in harsh underwater environment. Therefore, the TIG depositing technique may be applied in underwater construction instead of underwater repairs. For example, when joining the dissimilar steels (ferritic steel and austenitic stainless steel), the butter layer is prepared on the ferritic steel in the air before the steels was immersed into the water surroundings. The main advantages of butter layer are to develop an underwater wet dissimilar steel joint with improved mechanical properties, low residual stress and limited element diffusion.

The accelerated cooling rate experienced in the underwater environment results in the formation of quenched martensite in the HAZ of ferritic steel. The presence of the martensite layer leads to a high susceptibility to HIC. This could be proved by the existence of cracks in the HAZ in Figure 5a. The study demonstrated that the introduction of ERNiCrMo-3 butter layer with a favorable thickness effectively mitigated the presence of the hardened martensitic layer, hence enhancing the mechanical properties of underwater dissimilar joints. According to the previous results, the disappearance of hardened martensite in HAZ also was accompanied by the mitigation of tensile stresses in HAZ of the ferritic steel side. This result also has been obtained by Taraphdar et al. [16]. More importantly, the total alloying elements in weld metal increased, which strengthened the weld metal with the preparation of ERNiCrMo-3 butter layer. All in all, the application of the TIG depositing technique can be used to improve the mechanical properties of underwater DSWJ.

## 4. Conclusions

This study presented the effects of ERNiCrMo-3 layer on the element distribution, microstructural evolution, and tensile properties of underwater wet 16Mn/304L dissimilar steel joints. The main conclusions were as follows:

1. A gradient distribution of elements in each layer from the fusion boundary of 16Mn side to the nickel-based weld metal was clearly observed as the butter layer thickness increased. Fe content decreased gradually, while Ni and Cr contents increased until stabilized in the nickel-based weld metal. In the nickel-based weld metal, Fe content decreased progressively while Ni and Cr contents increased until they stabilized.
2. As the thickness of butter layer increased, HAZ in the dissimilar steel underwater wet welding process shifted from the original 16Mn side to the ERNiCrMo-3 interlayer, resulting in a gradual reduction of martensite content in the HAZ of 16Mn side. The introduction of butter layer enhanced the ultimate tensile strength from 515 MPa to 565 MPa, which surpassed the tensile strength of the 16Mn steel base metal.
3. The main advantages of butter layer were to improve mechanical properties, decrease residual strain and control the element diffusion. Future research should be focus on the residual stress distribution, fatigue properties and corrosion properties of underwater wet DSWJs. These findings would encourage additional research to manufacture high-quality underwater wet welded joints.

## Figures and Tables

**Table 1:** Charpy impact toughness of underwater DSWJ at different butter layer thickness.

Butter Layer Thickness (mm)	Impact Toughness		
	HAZ at 16Mn Side (J/cm^2^)	Weld Metal (J/cm^2^)	Butter Layer (J/cm^2^)
0	39	140	-
2	45	146.3	-
4	81.3	152.6	-
6	83.1	168	100
8	89	164.7	98

## Data Availability

The raw data generated during the present study are available from the corresponding author on reasonable request.
